# Role of stilbenes against insulin resistance: A review

**DOI:** 10.1002/fsn3.2553

**Published:** 2021-09-13

**Authors:** Shazmeen, Iahtisham‐Ul Haq, Muhammad Shahid Riaz Rajoka, Muhmmad Asim Shabbir, Muhammad Umair, Inam‐u llah, Muhammad Faisal Manzoor, Arash Nemat, Muhammad Abid, Moazzam Rafiq Khan, Rana Muhammad Aadil

**Affiliations:** ^1^ National Institute of Food Science and Technology University of Agriculture Faisalabad Pakistan; ^2^ School of Food and Nutrition Faculty of Allied Health Sciences Minhaj University Lahore Pakistan; ^3^ Food and Feed Immunology Group Laboratory of Animal Food Function Graduate School of Agricultural Science Tohoku University Sendai Japan; ^4^ Department of Food Science and Engineering College of Chemistry and Engineering Shenzhen University Shenzhen China; ^5^ Department of Food Science and Technology The University of Haripur Khyber‐Pakhtunkhwa Pakistan; ^6^ School of Food and Biological Engineering Jiangsu University Zhenjiang China; ^7^ Riphah College of Rehabilitation and Allied Health Sciences Riphah International University Faisalabad Pakistan; ^8^ Department of Microbiology Kabul University of Medical Sciences Kabul Afghanistan; ^9^ Institute of Food and Nutritional Sciences Arid Agriculture University Rawalpindi Pakistan

**Keywords:** biological pathways, insulin resistance, resveratrol, type 2 diabetes mellitus

## Abstract

Insulin resistance (IR) is a state characterized by the inability of tissues to utilize blood glucose particularly liver, muscle, and adipose tissues resulting in hyperglycemia and hyperinsulinemia. A close relationship exists between IR and the development of type 2 diabetes (T2D). Therefore, therapeutic approaches to treat IR also improve T2D simultaneously. Scientific evidence has highlighted the major role of inflammatory cytokines, reactive oxygen species (ROS), environmental & genetic factors, and auto‐immune disorders in the pathophysiology of IR. Among therapeutic remedies, nutraceuticals like polyphenols are being used widely to ameliorate IR due to their safer nature compared to pharmaceutics. Stilbenes are considered important metabolically active polyphenols currently under the limelight of research to cope with IR. In this review, efforts are made to elucidate cellular and subcellular mechanisms influenced by stilbenes including modulating insulin signaling cascade, correcting glucose transport pathways, lowering postprandial glucose levels, and protecting β‐cell damage and its effects on the hyperactive immune system and proinflammatory cytokines to attenuate IR. Furthermore, future directions to further the research in stilbenes as a strong candidate against IR are included so that concrete recommendation for their use in humans is made.

## Graphical abstract

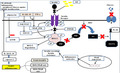

## 1 INTRODUCTION

Insulin, a peptide hormone secreted by the endocrine region of the pancreas in which β‐cells are responsible for their production in response to high blood glucose levels, modulates the availability of nutrients to target cells. In insulin resistance (IR), there is a decrease in the sensitivity of insulin receptors toward the insulin, and this abnormal behavior of the cellular insulin receptors results in hyperglycemia and hyperinsulinemia. Overproduction of insulin causes an increased workload leading to β‐cells decompensation, a major mechanism in the development of type 2 diabetes (T2D) (Taylor, 2012). T2D characterized by hyperinsulinemia, hyperglycemia, polydipsia, polyphagia, polyuria, unintentional and sudden weight loss, blurred vision, frequent infections, tiredness, and slow healing of sores (DeFronzo et al., 2015). These symptoms can lead to severe complications like CVD's, stroke, blindness caused by diabetic retinopathy, amputation due to poor blood flow to the limbs, and kidney disease (Pasquel & Umpierrez, 2014). The most known causes of insulin‐resistant or diabetes mellitus may include genetic influences, obesity, various environmental factors, high consumption of processed and refined foods, and being physically inactive (Duque‐Guimarães & Ozanne, 2013; Shah et al., 2021).

The prevalence of T2D has become a global pandemic during the last few decades rapidly increasing in low and middle‐income countries (McMurry et al., 2019). The estimated cases of diabetes have increased sharply worldwide. In 1980, there were 108 million estimated cases globally, while in 2014 these cases were mounted to 422 million, and the global prevalence of diabetes among adults has increased from 4.7% to 8.5% during this period. International Diabetes Federation (IDF) reported that currently, there are about 451 million adults having diabetes worldwide and 2,045 will be about 693 million if this trend continues. China has the highest number of diabetic patients approximately 144.4 million, while India ranks second and the USA third with 72.9 and 30.2 million cases, respectively. India will be at the top by 2045 with estimated cases of 134.3 million. Pakistan ranks at tenth position with 7.5 million diabetic patients which can be up to 16 million in 2045 and taking it to number 8 in the international ranking for diabetic burden (Control & Prevention, 2020).

Up till now, several chemotherapeutic/pharmaceutical agents have been approved by FDA for the management of T2D. Metformin is the most prescribed medication for T2D. It is very safe and inexpensive and can reduce A1C level 1 to 1.5%. Metformin can reduce glucose synthesis by the liver and is also cardio‐protective (Strack, 2008). Sulfonyl urea is another class of medication including glipizide, glyburide, and glimepiride used in the treatment of T2D, but Sulfonyl urea can cause hypoglycemia and weight gain if used for a prolonged time. Orlistat which is an antihyperlipidemic drug is a gastric and pancreatic lipase inhibitor. It has also shown remarkable results against T2D and has been effectively used in the treatment of T2D, but its long‐term use can cause subacute liver toxicity and steatorrhea (Walter et al., 2018). Rosiglitazone is an insulin‐sensitizing agent having side effects like upper respiratory track infection, head ache, and back pain. Other classes of drugs used in the T2D treatment plan include dipeptidyl peptidase‐4 inhibitors, an alpha‐glucosidase inhibitor, and sodium–glucose cotransporter‐2 inhibitors. These groups of medications have side effects including respiratory tract infections, joint pain, abdominal cramping, diarrhea, increased urination, and thirst (Han et al., 2021).

Polyphenols, a vast family of naturally occurring organic compounds present abundantly in plant‐based foods and are characterized by the presence of multiple phenolic rings in their structures, include about 8,000 different chemical compounds. Their main sources are fruits, vegetables, dry fruits, nuts, seeds, and roots, bark, leaves of different plants, herbs, whole grain products, processed foods and also sufficient quantity in tea, coffee, and red wine (Adebooye et al., 2018; Manzoor et al., 2017, 2019). Stilbenes, an important polyphenolic compound, possess several health benefits including angiogenesis, cell proliferation, mitochondrial activity, anti‐inflammatory, lipolysis of adipocytes, and redox status modulation. Stilbenes have been proved to protect against several chronic diseases like cardiovascular diseases, several types of cancers, neurodegenerative diseases and insulin resistance (IR), etc. (Kershaw & Kim, 2017). Stilbenes have shown significant results by improving IR and ameliorating T2D. Some common stilbenes sources include sorghum (Poaceae), peanut (Fabaceae), grape (Vitaceae), and pine (Pinaceae) (Dubrovina & Kiselev, 2017). Stilbenes are linked to many important physiological activities in the cell including induction of antioxidant enzyme system, inhibit the transcription of factors taking part in inflammatory pathways like nuclear factor kappa B (NF‐κB), mitogen‐activated protein kinases (MAPKs), and activator protein‐1 (AP‐1). Stilbene triggers adenosine monophosphate kinase (AMPK) which subsequently increases the glucose uptake in myocytes (Furtado et al., 2002; Yamaguchi et al., 2005). It can inhibit the accumulation of fat by downregulation of adipocyte‐specific proteins during differentiation of 3T3‐L1 cells (Jinfeng Li et al., 2018). In this review, relevant studies have been analyzed to summarize the various cellular and molecular pathways involved in IR ultimately leading to T2D. Also discussed in this review is the role of stilbenes against IR. Various mechanistic approaches have been elaborated to show the protective effect of stilbenes against IR.

## 2 PATHOLOGY OF IR

**FIGURE 1:**
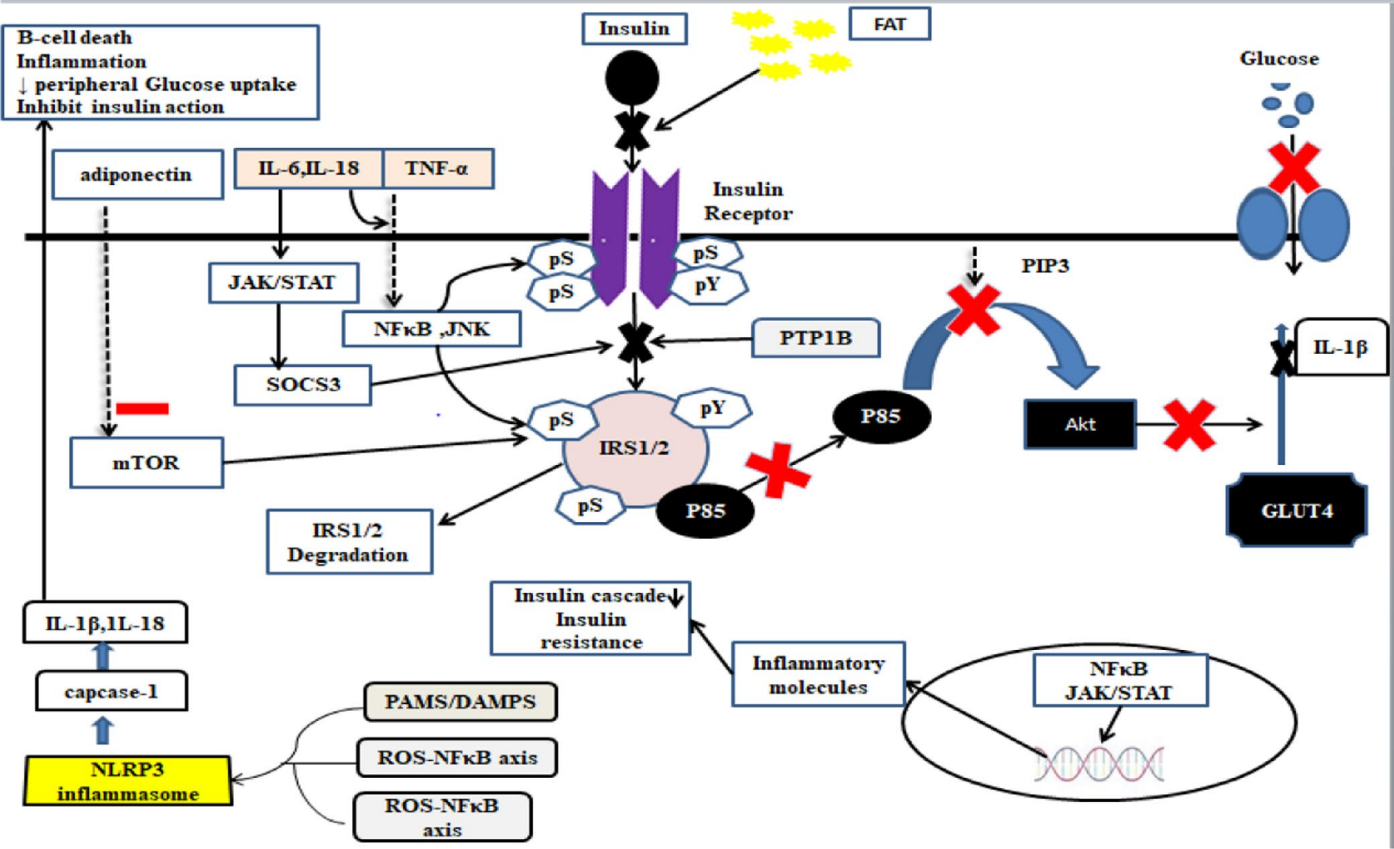
Pathophysiology of insulin resistance

IR is a common character of many metabolic disorders including dyslipidemia, metabolic syndrome, T2D, nonalcoholic fatty liver disease, obesity, atherosclerosis, hypertension, and polycystic ovary syndrome (Barazzoni et al., 2018; Diamanti‐Kandarakis & Christakou, 2009). The metabolic fate of IR is represented in Figure 1. The pathophysiology and metabolic markers influencing IR (Table 1) are discussed here in detail. PTP1B is a nontransmembrane enzyme that inhibits phosphorylation in tyrosine residues of Insulin Receptor Substrate 1 (IRS‐1) and so impairs insulin signal transduction. PTP1B also inhibits the activity of Insulin Receptor Kinase (IRK) by forming an inhibition complex by attaching to growth factor receptor‐bound protein 2 (GRB2) (Boute et al., 2003). PTP1B downregulation is linked to lower IR development while overexpression is a prime candidate for IR and obesity. In a rat model, the overexpression of PTP1B showed increase IR and weight gain while knocking down via PTPB1B antisense oligonucleotides resulted in improved insulin signaling (Wang et al., 2017). The primary candidates that are involved in the expression of PTP1B are ER stress and ROS. Study data suggest that PTP1B upregulation in ER stress is through the formation of ROS‐NF‐κB axis that causes inhibition of insulin action (Panzhinskiy et al., 2013).

**TABLE 1:** Physiological biomarkers influencing insulin resistance

Factors involved		Link with IR	References
PTP1B upregulation		Inhibits IRS−1 phosphorylation, impairs insulin signal transduction, ↑ weight gain, ↓ IRK	(Panzhinskiy et al., 2013)
Adipocyte hyperplasia and IR		↓ TNF‐α, leptin, adiponectin, plasminogen‐activator inhibitor−1, angiotensinogen, activate serine–threonine kinase thus inhibiting the insulin signaling cascade, ↑ JNK and NF‐ĸB, monocyte infiltration	(Castoldi et al., 2016; Lee & Lee, 2014; Reilly & Saltiel, 2017)
NLRP3 inflammasome		↑ Macrophages, NF‐ĸB upregulation, Procapacse−1 activation to capcase−1, ↑ IL−1β and IL−18	(Ballak et al., 2015; Davis et al., 2011; Shao et al., 2015)
Proinflammatory cytokines	IL−1β	β cells apoptosis, ↓insulin sensitivity, suppress expression of GLUT4, activation of NADPH oxidase produces superoxide anion	(Donath et al., 2019; Gao et al., 2014; Herder et al., 2015; Meyerovich et al., 2016)
	IL−18	Proinflammatory cytokine, systemic inflammation,↑ NF‐κB, P38, JNK, ERK, and ↑ STAT3/MAPK	(Osborn et al., 2008; Vallejo et al., 2014)
	IL−6	SOCS3 activation, ↓ tyrosine phosphorylation of IRSs IKB, ERK1, ERK2, activate JAK‐STAT3, ↓GLUT4 and PPAR‐γ, lipolysis	(Ciccarelli et al., 2016; Fève & Bastard, 2009; Gurzov et al., 2016)
	TNF‐α	Serine phosphorylation of IRS1, ↑ JNK and NF‐κB, systematic inflammation	
Innate and adaptive immunity	Macrophages	M1‐polarized macrophages release TNF‐α and IL−1β, activation of Kupffer cells, ↑ IL−6 and MCP−1	(Lee et al., 2018; Reilly & Saltiel, 2017)
	Neutrophils	Production of TNF‐α and MCP−1, produce peptide elastase, hinders glucose uptake in adipose tissues and destroying IRS1	(Kane & Lynch, 2019; Trim et al., 2018)
	Dendritic Cells	Production of IL−6 and IL−28, Recruitment of macrophage, ↓ GLP−1 and glucose‐dependent insulinotropic polypeptide	(Asghar & Sheikh, 2017; Chung et al., 2018; McLaughlin et al., 2017)
	Mast cells	Macrophage infiltration, ↑ histamine, chemokines, and cytokines	(Lackey & Olefsky, 2016)
	B cells	↑ Chemokines secretion, monocytes recruitment, T cells, and neutrophils, ↑ macrophages	(Dam et al., 2016; Winer et al., 2011)
	T Cells	Secretion of IFN‐γ, ↓ of anti‐inflammatory type of cells Th2 and (Tregs)	(Henao‐Mejia et al., 2014; Nishimura et al., 2009)
Dysregulated FA Homeostasis		SFA bind to TLR4 and TLR2, NF‐κB and JNK pathway activation, release of cytokines MCP−1 and TNF‐α	(Könner & Brüning, 2011; Reilly & Saltiel, 2017)
Adipocyte Hypertrophy, Hypoxia, and Death		↑Increased rate of adipocyte death and infiltration of macrophages, cellular hypoxia, expression of vascular endothelial growth factor etc.	(Choe et al., 2016; Heilbronn & Liu, 2014)
Mitochondrial dysfunction		Triggers inflammatory mechanisms like NF‐κB and inflammasome activation, cytokines and adhesion molecule upregulation, accumulation of intracellular fatty acyl‐CoA and DAG, blocking insulin signaling	(Cano Sanchez et al., 2018; Longo et al., 2019; Sergi et al., 2019)
Endoplasmic reticulum stress		↑ JNK, NF‐κB, apoptosis signaling pathways, production of inflammatory molecules like IL−8, IL−6 MCP−1 by endothelial cells	((Jeschke & Boehning, 2012)

The chronic imbalance that occurs in intake of energy and its expenditure results in overweight and obesity and an increased accumulation of fat in adipose tissues (Chooi et al., 2019). Adipocytes act as endocrine cells and release inflammatory mediators like TNF‐α, leptin, adiponectin, plasminogen activator inhibitor‐1 (PAI‐1), angiotensinogen, and active steroid hormones having the potential to induce IR (Halberg et al., 2008). High levels of plasma lipids also activate serine–threonine kinase thus inhibiting the insulin signaling cascade (Gills & Dennis, 2004). They also cause the activation of JNK and NF‐ĸB pathways. They enhance the level of proinflammatory cytokines, chemotactic mediators, and endothelial adhesion molecules, and there is an increased infiltration of monocytes into adipose tissues which then differentiate into M1 macrophages (B.‐C. Lee & Lee, 2014; Reilly & Saltiel, 2017). In case number of adipose tissues is high, macrophages in these adipose tissues start to show a proinflammatory phenotype and release several proinflammatory cytokines which result in inflammation and IR (Castoldi et al., 2016). These macrophages produce proinflammatory cytokines and chemokines that result in local and systematic inflammation (Burhans et al., 2011; Haase et al., 2014).

Inflammasomes of many types have been identified including NLRP1, NLRP2, and NLRP3. The inflammasome pathway is activated by myeloid cells in obesity (Vandanmagsar et al., 2011). NLRP3 activated by Danger Associated Molecular Patterns (DAMPs) has a crucial role in chronic inflammation and IR (Shao et al., 2015). Activation of NLRP3 that interacts with procaspase‐1 by adaptive protein results in the NLRP3 inflammasome formation, which causes capcase‐1 activation. Capcase‐1 results in macrophages maturation and release of IL‐1β and IL‐18 (Ballak et al., 2015). Furthermore, NLRP3 gene aberration results in the prevention of obesity‐induced inflammation (Böni‐Schnetzler et al., 2009).

IL‐1β is a proinflammatory cytokine and is an important agonist of pancreatic β‐cell death in T2D (Ortis et al., 2008). IL‐1β decreases insulin‐stimulated glucose uptake and lipogenesis via decreasing the expression of GLUT4 and its plasma membrane translocation (Gao et al., 2014). IL‐1β binds to the cognate receptors present on β‐cells and triggers signaling cascade which triggers intracellular signaling pathways by recruiting adaptor proteins and kinase receptors. As a result, NF‐κB and MAPK signaling cascades are activated (Meyerovich et al., 2016). IL‐1β has also been linked to complications of diabetes mellitus like retinopathy, nephropathy, and cardiovascular complications (N. K. Agrawal & Kant, 2014). IL‐1β causes NADPH oxidase activation by IL‐1R leading to ROS production, which causes atherosclerosis development in diabetic patients (Herder et al., 2015). IL‐18 is a strong proinflammatory cytokine that causes systemic inflammation (Vallejo et al., 2014). IL‐18, after its release and activation, attaches to the IL‐18 receptor (IL‐18R) which engages myeloid differentiation primary response gene 88 (MyD88), and MyD88 through mediators sends an activation signal to IĸB kinases (ikks) causes the release of NF‐ĸB which then mediate inflammatory responses leading to IR (Osborn et al., 2008). IL‐18 release and activation have also been linked to p38 release and upregulation of STAT3/MAPK signaling cascade (S. Agrawal et al., 2011). These intracellular effects produced by 1L‐18 make it a powerful target to prevent IR and T2D. IL‐6 is responsible for the macrophage recruitment into the adipocytes thus producing inflammation there. It also inhibits the action of insulin on adipocytes preventing their lipolysis and induces apoptosis in β‐cells (Akbari & Hassan‐Zadeh, 2018). IL‐6 action mechanism involves the Janus kinase (JAK)‐signal transducer and activator of transcription (STAT) activation (Gadina et al., 2018). The JAK endorses serine phosphorylation in IRS‐1 and so blocks insulin action. Adipose tissue‐specific JAK2 KO mice had shown faulty lipolysis, adiposity, and increased weight leading to IR (Gurzov et al., 2016). Intracellular JAK‐STAT3 activated pathway causes increased expression of cytokine signaling‐3 suppressor (Ciccarelli et al., 2016). It causes impaired insulin signaling mainly via insulin‐stimulated tyrosine phosphorylation of IRSs by Suppressor of Cytokine Signaling 1 (SOCS1) and SOCS3 activation in adipose tissues and liver cells (Fève & Bastard, 2009). SOCS3 inhibits the insulin‐stimulated phosphorylation of IR‐β, IRS1, PKB, extracellular signal regulated kinase 1 (ERK1), and extracellular signal regulated kinase 2 (ERK2). This causes a downfall in the inulin signaling cascade leading to IR (Torisu et al., 2007). Moreover, SOCS3 also lowers the expression and translocation of GLUT4 and PPAR‐γ. TNF‐α is a potential candidate for IR induced T2D via inhibiting insulin‐induced tyrosine phosphorylation of IRS1 by serine phosphorylation of IRS1 and hence limiting insulin‐stimulated glucose uptake (Kampmann et al., 2011; Swaroop et al., 2012). TNF‐α binding to its receptor sites results in the onset of signaling cascades which subsequently causes activation of two main inflammatory pathways JNK and NF‐κB (Subedi et al., 2017).

Saturated FAs have a strong link to promote an inflammatory response in the body through activation of macrophages (Funaki, 2009). They activate macrophages due to indirect attachment to TLR4 and TLR2, which subsequently causes the stimulation of NF‐κB and JNK pathway, and these stimulated pathways trigger the release of MCP‐1 and TNF‐α, resulting in adipose infiltration of macrophages and causing IR (Könner & Brüning, 2011; Reilly & Saltiel, 2017). Adipocyte hypertrophy triggers the activation and infiltration of macrophages into adipocytes, and these macrophages attached to necrotic adipocytes (Heilbronn & Liu, 2014). These monocytes have the ability to produce inflammatory cytokines and ROS in nearby adipocytes which induce IR in them (Choe et al., 2016).

Due to obesity and high level of plasma lipids, degradation can occur in the function of mitochondria which results in metabolic dysfunction, oxidative stress, inflammation, cell death, and IR (Montgomery & Turner, 2015). There has been observed a decrease in mitochondrial activity and reduction in DNA of mitochondria in obese mice and in obese humans (Yin et al., 2014). The malfunctioning in mitochondria triggers inflammation via inflammasome activation and NF‐κB pathway, which causes inflammatory cytokines upregulation and adhesion molecule secretion and a significant increase in inflammation and IR (Cano Sanchez et al., 2018; López‐Armada et al., 2013).

ER stress can be significantly increased by obesity mainly in the liver and adipocytes (Gregor et al., 2009). ER stress negatively affects metabolic homeostasis chiefly through induction of inflammation (Cinti et al., 2005). ER stress establishes inflammatory responses in adipocytes via NF‐κB and JNK activation and stimulation of apoptosis signaling pathways (Sergi et al., 2019). Dysfunction of unfolded protein response (UPR) causes NF‐κB activation with insulin action inhibition through IRS1 phosphorylation (Longo et al., 2016).

## 3 PHOTOCHEMISTRY OF STILBENES

**FIGURE 2:**
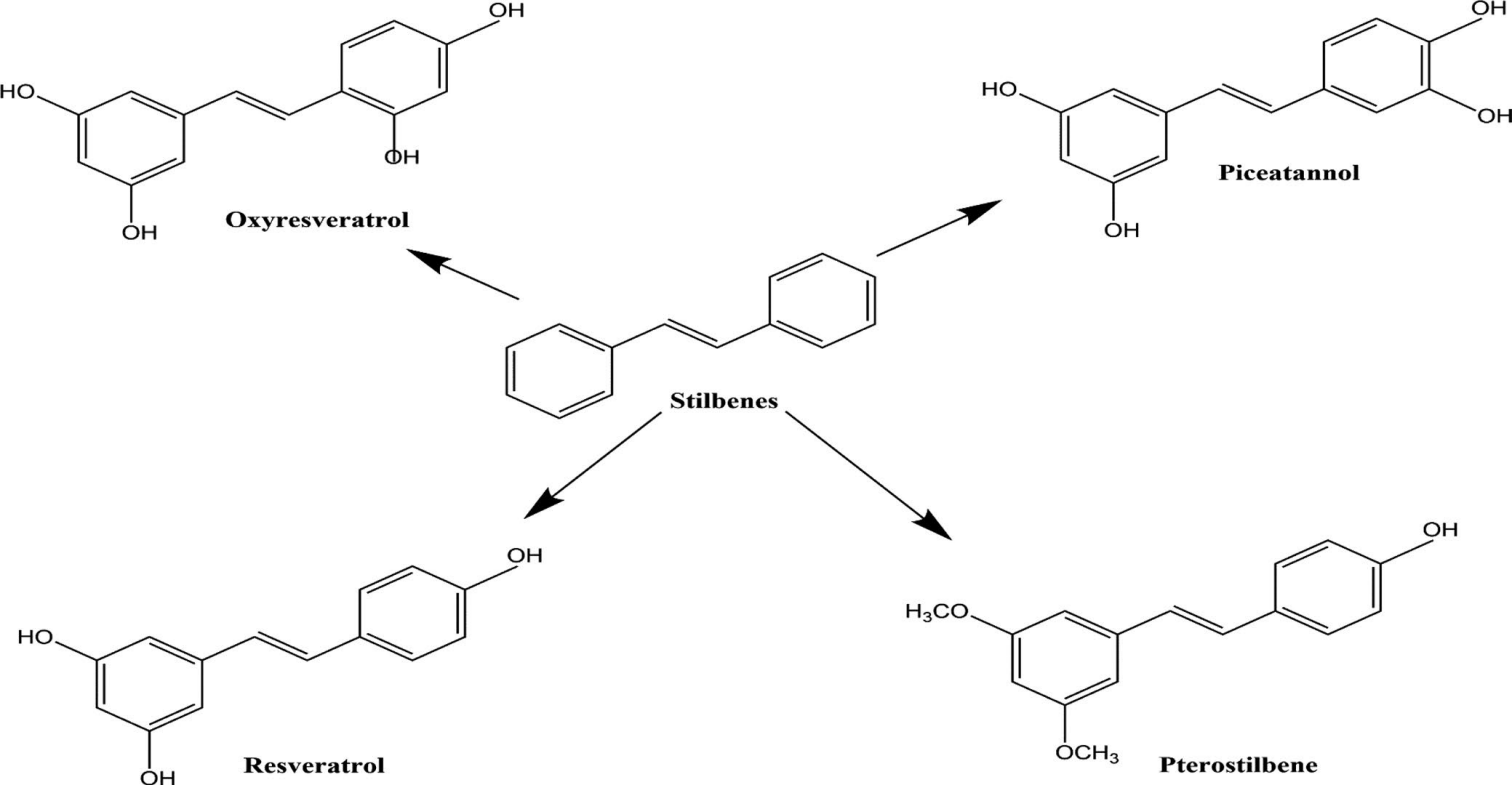
Chemical structures of some important stilbenes

Stilbenes are an important group of secondary metabolites belonging to a subclass of phenylpropanoids that responds to various biotic and abiotic hazardous environmental factors and protects plants. Some important stilbene compounds have been illustrated in Figure 2. The name stilbene was derived from “Stilbos” meaning shining and is considered as phytoalexins. Stilbenes have 1, 2‐diphenylethylene backbone and consist of two aromatic rings of phenyl group linked by ethylene bridge. They are mostly derivatives of transresveratrol and up to 400 derivatives have been identified till now. Stilbenes have two isomeric forms: (E)‐stilbene (trans‐stilbene), which is more stable and is not sterically hindered, and (Z)‐stilbene (cis‐stilbene), which is less stable and is sterically hindered. Some biologically active trans‐stilbenes include oxyresveratrol, pterostilbene, piceatannol, isorhapotigenin, *cis*‐stilbene, and resveratrol (3,4,5‐trihydroxystilbene) (Shen et al., 2009).

**FIGURE 3:**
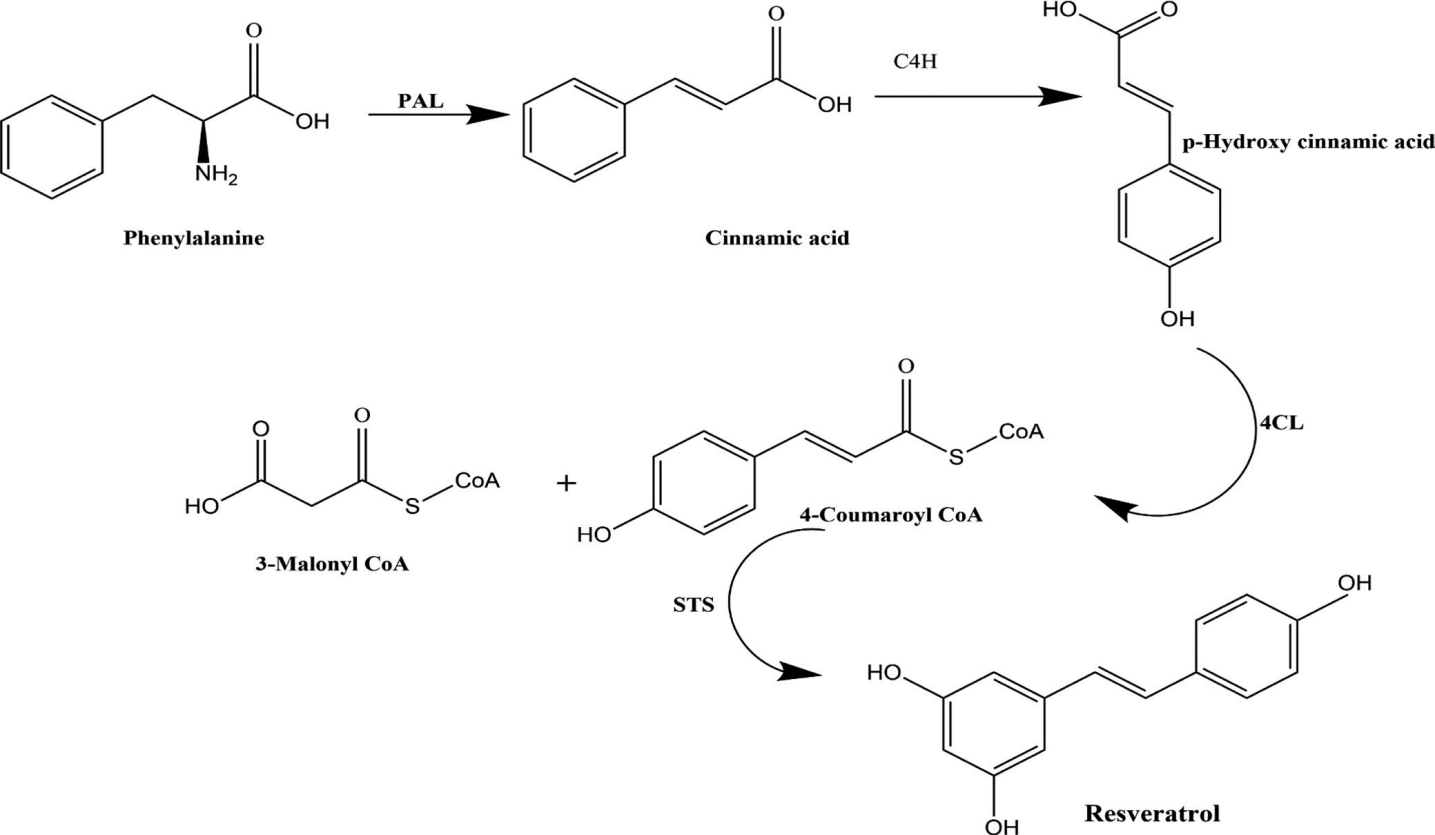
Biosynthesis of stilbenes (resveratrol)

Biosynthesis of stilbene begins by the phenylpropanoid pathway (Figure 3). Initially, phenylalanine ammonia‐lyase (PAL) converts L‐phenylalanine into transcinnamic acid which is further isomerized into p‐coumaric acid by tyrosine ammonia‐lyase (TAL). In the very next step, 4‐coumarate‐CoA ligases (4Cl) transforms transcinnamic acid or p‐coumaric acid into cinnamoyl‐CoA or p‐coumaroyl‐CoA. Then, the first critical step of stilbenes formation takes place in which stilbene synthase catalyzes the malonyl‐CoA and CoA‐ester of cinnamic acid derivatives (cinnamoyl‐CoA, p‐coumaroylCoA, or caffeoyl CoA) for the synthesis of monomeric stilbenes (Dubrovina & Kiselev, 2017).

Modifications in these monomeric stilbenes like methylation, glycosylation, oligomerization, and prenylation to convert them into various kinds of stilbenes. These modifications are controlled by various enzymes; an important enzyme of this pathway that is directly involved in stilbene synthesis is stilbene synthase (STS) (Jeandet et al., 2010). Examples of some common stilbenes derived from different plant sources are enclosed in Table 2.

**TABLE 2:** Concentration of stilbenes in different plant and plant parts

Plant name	Plant part	Quantities	Type of stilbene	References
Red sorghum	Grain	0.4–1 mg/kg	*Trans*resveratrol *Trans*‐Piceid	(Bröhan et al., 2011)
Transgenic Arabidopsis	Whole plant extract	10‐µg/g	Resveratrol diglucoside *Trans*‐ and *cis*‐resveratrol acetylhexosides	(Lo et al., 2007)
Peanut(Arachis hypogaea)	Cotyledons raw and roasted	75‐µg/ounce	*Trans*‐ and *cis*‐resveratrol	(Sanders et al., 2000)
	Peanut skin	9.7‐µg/g	Resveratrol hexoside	((Nepote et al., 2005)
	Peanut leaves	2.05‐μg/g	3,5,4′‐trihydroxystilbene	(S.‐Y. Chung & Champagne, 2009)
	Peanut roots	1.19‐μg/g	Resveratrol *t*‐arachidin−1 *t*‐arachidin−2 *t*‐arachidin−3	(Lopes et al., 2013)
Grapes	Skin	10.97‐μg/g	*Trans*resveratrol Vaticanol C‐like isomer Cis‐piceid *Trans*piceatannol α‐viniferin	(Gil‐Muñoz et al., 2017)
	Wine	0.45‐μg/g	*Trans*resveratrol Ampelopsin D *Trans*isorhapontigenin	(Gil‐Muñoz et al., 2017)
	Grape cane	6,915 ± 175‐μg/g	*Trans*resveratrol *Trans*‐ε‐viniferin *Trans*piceatannol	(Vergara et al., 2012)
Pine	Heart wood	0.2%–2%	Pinosylvin Pinosylvin monomethyl ether	(Hovelstad et al., 2006)
	Living nots	2%–8%	Stilbenoids Dihydropinosylvin monomethyl ether Pinosylvin monomethyl ether	(Hovelstad et al., 2006)

## 4 BIOLOGICAL EFFECTS OF STILBENES AGAINST IR

Considering the biological markers involved in the pathophysiology of the stilbenes, the following discussion highlights the importance of stilbenes as a possible therapeutic agent against T2DM and/or IR. The summary of the effects of stilbenes on these biomarkers and their mechanisms are enclosed in Table 3 as well.

**TABLE 3:** Biological effects of stilbenes against IR

Biomarkers	Subject		Doses and duration	Effects	References
Protein‐tyrosine phosphatase 1B	In vitro assay		3.8, 7.0, and 7.6 µg/ml	PTP1B inhibition	((Na et al., 2008)
Adipocyte hyperplasia	Female C57BL/6 mice		5 g RES/kg for 11 weeks	↓ Stem cell differentiation to preadipocytes, ↑ in lower pathogenic in adipocytes, ↓ mammary tissue PPARγ expression, adipogenic, fibroblast growth factor 2, nuclear receptor subfamily 0 group B member	(Rossi et al., 2018)
	3T3 F442A cells from male Swiss mice		20 µM RES for 8 days	↓ α‐glucosidase and lipase activity, inhibits insulin stimulated adipogenesis, ↓ insulin induced glucose uptake in adipocytes, ↓ lipogenesis and antilipogenic effects	(Carpéné et al., 2019)
	Male OLETF rats		300 mg kg^−1^ day^−1^ pterostilbene for 3 weeks	↑SIRT1 and PPARα, suppressed the abdominal WAT accumulation by 25% ↑ Fat oxidation, ↑ UCP2 mRNA expression	(Nagao et al., 2017)
Dysregulated Fatty Acids Homeostasis	Wild‐type zebrafish (Danio rerio)		20 μmol/L for duration of 8 weeks	↓ plasma TG, relieved the destroyed hepatic structure, ↑ AMPKα activity and expression of Sirt1, ↓ PPARγ, ↓ total cholesterol levels	(Ran et al., 2017)
	Male Wistar rats		20 mg kg^−1^ day^−1^ for duration of 8 weeks	↓ TGS, CHOL LDL, and VLDL, ↓ Accumulation of fat droplets, ↓ lipogenic genes (FAS), ↑ HMG‐CoA reductase), GSH. SOD	(Khaleel et al., 2018)
NLRP3 inflammasome	Cultured mouse podocytes (MPC5)		Tetrahydroxy stilbene glucoside TSG (0.1, 1, 10 μM) for 48 hr	↓ NLRP3 inflammasome expression ↓ IL−1β, ↓ caspase−1 expression, ↑ Nephrin, Inhibited oxidative stress	(Li, Wang, et al., 2018)
	adult male C57BL/6 J mice		10 mg kg^−1^ day^−1^ pterostilbene for 24, 72 hr	↓ ASC, caspase−1 p20, IL−1β, and IL−18, attenuates Oxidative Stress Reduced NLRP3 inflammasome activation	(Liu, Zhao, et al., 2017)
Proinflammatory cytokines	IL−1β	Chondrocyte monolayer cultures	50 μM resveratrol, 50 μM curcumin or cotreatment for 4 hr	↓ NF‐κB activation, ↓ cytotoxic effects of IL−1β, ↑ Bcl−2, Bcl‐xL, and TRAF1 (antiapoptotic proteins), IκBα ↑	(Csaki et al., 2009)
	IL−18	Human aortic SMCs	25 μM for 1 hr	↓ Blocks IL−18 signaling, Akt kinase activity, ↓ IL−18‐mediated ROS generation, ↓ IL−18‐mediated PI3K activity, ↓ ERK activity	(Venkatesan et al., 2009)
	IL−6	NIH‐OVCAR3 ovarian cancer cells	100 µM for 72 hr	Inhibits STAT3 activation via IL−6, ↓ STAT3, inhibits the activity of IL−6	(Ferraresi et al., 2017)
		Bovine aortic endothelial cells	100 μmol/L for 12 hr	↓ ICAM−1 gene expression, ↓ phosphorylation of STAT3, ↑ NO production, ↓ NADPH oxidase activities, Inhibits IL−6 activity	(Rochfort & Cummins, 2015)
	TNF‐alpha	Adult male Sprague‐Dawley rats	100 μmol/L, i.v 5 min before reperfusion	Reduced TLR4 protein expression, ↓ NF‐κB expression, reduced TNF‐α level	(Wang et al., 2017)
Innate and adaptive immunity	Macrophages	Female C57BL/6 mice	100 mg/kg of RES 6 hr before and 2 hr after treatment	↓ TNFα, MCP−1, IL−1β, and IL−6 ↑ Reduce immune cells infiltration into the kidney, IL−10, ↓ macrophage apoptosis and activation, ↓ iNOS, ↓ TNFα	(Wang et al., 2017)
	Neutrophils	Male Lewis rats	30 mg/kg pterostilbene for 21 days	↓ Neutrophil ROS production, ↓ number of neutrophils and neutrophil activity, ↑ the total peroxyl radical trapping capacity of plasma ↓ Neutrophil NADPH oxidase activation	(Perecko et al., 2013)
	Dendritic Cells	Male BALB/c and C57BL/6 mice	10 – 200 μg/mL concentrations for 24 hr	↑ Cytotoxic effect on DCs, CD86, and MHC‐II on DCs, ↑ IL−10 cytokine	(Shahnazi et al., 2015))
	Mast cells	Male ICR mice	1, 10, 50, and 100 μM conc. Of Piceatannol for 30 min	↓ Histamine release, ↓ TNF‐α and IL−8 gene expression, ↑ JNK MAPK activation, mast cells counter effect	(Ko et al., 2013)
	T cells	BALB/c and C57BL/6 female mice	25–75 µM of Resveratrol for 24 hr	↓ CD4+CD25+ Treg cell, reduced TGF‐β, ↓ IFN‐γ expression	(Yang et al., 2008)
	B cells	Human malignant B cell lines	50 μM RES for 48 hr	Inhibited malignant B cell proliferation, ↓ cycle arrest in B cells, ↑ caspase−3 activity, apoptosis‐associated proteins, ↑ p38 MAP kinase	(Shimizu et al., 2006)
Endoplasmic reticulum stress	C57BL/6 mouse model		(5 and 50) mg/kg BW for 3h, 1, 4, and 7 days	↓ Eukaryotic translational initiation factor 2α eIF2α, ↓ immunoglobulin binding protein (Bip), ↓ DNA damage‐inducible protein 34	(Jiong Li et al., 2012)
	Albino rats		20 mg kg^−1^ day^−1^ for 3 weeks	↓ Caspase−3 activity, ↓ IL−1β, ↑ Nrf2 DNA‐binding activity	(Gaballah et al., 2016)
Mitochondrial dysfunctioning	Neonatal male piglets		1 g/kg of milk dry matter for 7 to 21 days	↑ SIRT1, ↓ Mitochondrial DNA content and FA oxidation improved Superoxide anions	(H. Zhang et al., 2017)
	Primary hippocampal astrocyte cultures from male Wistar rats		100 μM of resveratrol for 1 hr	↓ TNF‐α, ↑ cytochrome c oxidase, ↑ HO−1, ↑ SOD1, SOD2, CAT, ↓ IL−1β	(Bellaver et al., 2016)

### 4.1 Inhibition of PTP1B

Inhibition of PTP1B can be a promising therapeutic target in the treatment of IR (Liu et al., 2017). A study conducted by Ha et al. (2018) indicated a positive association of stilbenes to inhibit PTP1B activity. In this study, ligand C and ligand A in silico molecular docking simulations were used to check the effects of stilbenes. Ligand C was stably positioned on the catalytic site of PTP1B and the hydrogen bonding between PTP1B and ligand C made a strong enzyme‐substrate complex that blocked the activity of PTP1B. The inhibition effect of stilbenes on PTP1B was dose‐dependent and showed positive results at a concentration of 30µg/ml. J. Li, Wang, et al. (2018) indicated that the binding of ligands in the α3 and α7 helices in PTP1B caused allosteric inhibition. J.‐B. Yang, Ye, et al. (2020) assessed the inhibitory effect of four stilbenes derived from *P. multiflorum* on PTP1B. Results indicated glycosylated stilbenes were potent inhibitors of PTP1B and could be potential future therapeutics for T2D treatment.

### 4.2 Control of adipocyte hyperplasia

Several stilbenes have been used to prevent and treat adipocyte hyperplasia including resveratrol (3,5,4′‐trihydroxy‐trans‐stilbene), oxyresveratrol (2,4,3′,5′‐tetrahydroxy‐trans‐stilbene), pterostilbene (3,5‐dimethoxy‐4′‐hydroxy‐trans‐stilbene), and piceatannol (3′,4′,3,5‐tetrahydroxy‐trans‐stilbene) (Reinisalo et al., 2015). The basic study design used to assess the effect of these stilbenes was 3T3‐L1 preadipocyte. Resveratrol abundantly present in blueberries, grapes, and peanuts, can inhibit adipogenesis and lowers fat accumulation in the 3T3‐L1 preadipocyte mouse model (Rayalam et al., 2008). Kwon et al. (2015) indicated resveratrol at a concentration of 50 μM lowered fat accumulation up to 55% and also declined the PPARγ and C/EBPα levels. Resveratrol also declined the expression of Cyclin A and cyclin‐dependent kinase 2 (CDK2). In a study, treatment with 30µM of resveratrol results in downregulation of SREBP‐1c and its target gene FAS is validated by Oil Red O staining performed on fat accumulated intracellular hepatocytes. Resveratrol remarkably changes the fat accumulation in the cell (Jin et al., 2013). Oxyresveratrol occurs commonly in white mulberry and *Artocarpus lakoocha*. Oxyresveratrol prevents the differentiation of preadipocytes by retaining them in the G1 phase (Tan et al., 2015). Data from human genetic studies and in rat models indicated that a reduction in PPARγ has positive association with improved insulin sensitivity (Miles et al., 2000; Picard & Auwerx, 2002). In a study, 100 μM oxyresveratrol reduced triglyceride accumulation and lowered PPARγ and C/EBPα expression by modulating cyclin D1 and cyclin‐dependent kinase 4 (CDK4). The scientists concluded that oxyresveratrol has role in decreasing triglyceride accumulation and cell cycle arrest in adipocytes which improves insulin signaling pathway and lowering IR. Moreover, it also downregulates the expression of PPARγ thus ameliorating insulin resistance (Tan et al., 2015). In adipose tissues, oxyresveratrol upregulates GLUT4 and promotes glucose and fatty acid catabolism (Chooi et al., 2019).

Piceatannol occurs abundantly in grapes, passion fruit, Japanese knotweed, Asian legume, and blueberries (Banik et al., 2020). It inhibits the adipogenesis of preadipocytes (Kwon et al., 2015). In a study, it was found that 25–50 µM of piceatannol decreases serum cholesterol, decreases body weight, and lowering the LDL/HDL ratio. It also has a vital role in alleviating obesity‐induced inflammation in adipocytes which otherwise can lead to metabolic syndromes (Yang et al., 2020). Yamamoto et al. (2015) conducted a study that indicates that supplementation of piceatannol at a concentration of 30 μM curbed inflammatory cytokines TNF‐α and IL‐6. Similarly, Yanfang Li, Yang, He, et al. (2017) indicated that piceatannol at a concentration of 10 μM decreases the level of TNF‐α and monocyte chemoattractant protein‐1 (MCP‐1).

Pterostilbene naturally occurs in raspberries, blueberries, mulberries, grapes, and peanuts (Chan et al., 2019). Pterostilbene is dimethyl ether analogue of resveratrol. Pterostilbene in 3T3‐L1 preadipocytes induces cell apoptosis and inhibits adipogenesis which results in lower fat accumulation (Hsu et al., 2012). It has been observed that 6 μM of pterostilbene can decrease lipid accumulation by suppressing adipogenesis. Pterostilbene has an inhibitory effect on C/EBPα and PPARγ expression (Rossi et al., 2018; Seo et al., 2017).

### 4.3 Inhibition of NLRP3 inflammasome activity

Stilbenes can inhibit NLRP3 inflammasome activity and preventing its role in IR development through different mechanisms and pathways. Stilbenes have been shown to suppress the activation of macrophage activation and thus inhibiting the NLRP3 inflammasome activation (G. Li, Wang, et al., 2018). Another pathway for NLRP3 inflammasome inhibition involves SIRT1 activation and induction of autophagy. Resveratrol is a potential activator of SIRT1 which prevents the acetylation of primary inflammatory molecules (Borra et al., 2005). In a study, Lagouge et al. (2006) indicated that 10 μM resveratrol inhibited the NLRP3 inflammasome by inducing autophagy through activation of SIRT1. Mitochondrial damaged induced by inflammasome is also suppressed by resveratrol treatment via inducing apoptosis in them (Sareen et al., 2006). Inhibitory role of resveratrol on NLRP3 inflammasome has been observed in peritoneal mesothelial cells, mesenchymal cells, and renal epithelial cell line cells. TXNIP inhibition is another inflammasome inhibitory pathway. In different in vivo models, inhibition of TXNIP by resveratrol leads to NLRP3 inflammasome suppression (Olcum et al., 2020).

### 4.4 Modulation of proinflammatory cytokines

One of the most extensively highlighted mechanisms involved in IL‐1β activation and release is capcase‐1. Capcase‐1 is itself activated through NOD‐like receptor (NLR) driven ASC cleavage of procapcase‐1, which then recruits pro‐IL‐1β to produce IL‐1β. NLRs include NLRP1, NLRP3, NLRP6, NLRP7, NLRP12, and NLRC4, which are all involved to induce IL‐1β production (Zhang et al., 2018). Stimulation by pathogen‐ and/or danger‐associated molecular patterns (PAMPs/DAMPs) incites the production of IL‐1β chiefly via NLRP3 activation (He et al., 2016). Thus, NLRP3 inflammasome suppression is an important target to impede IL‐1β activation and attenuate T2DM related IR. Studies have confirmed the role of stilbenes (resveratrol) in the activation of IL‐1β via suppression of NLRP3 (Zou et al., 2018). Resveratrol is an activator of silent information regulator 1 (Sirt1), which is a NAD +‐dependent class III histone deacetylase. SIRT1 is a potential down regulator of NLRP3 expression. Thus, resveratrol inhibits the expression of IL‐1β via SIRT1 mediated suppression of NLRP3 (Li, Yang, He, et al., 2017).

IL‐6 is an important inflammatory cytokine mainly released by adipose tissues. IL‐6 is also involved in the auto‐activation of inactive IL‐6. Once IL‐6 activates, it attaches to the IL‐6 receptors (IL‐6R). IL‐6R can exist in both attached to membranes (mbIL‐6R) or insoluble form (Sil‐6R), binding either of them results in inactivation of the intracellular inflammatory cascade (Akbari & Hassan‐Zadeh, 2018). IL‐6 then induces the intercellular adhesion molecule (ICAM‐1) expression via the NF‐κB and JAK/STAT3 pathways which further causes STAT3 phosphorylation on tyrosine 705 (Dziemidowicz et al., 2019). IL‐6 also caused upregulation of suppressor of cytokine signaling (SOCS3) expression in Hep G2 cells and in rat liver cells which results in impaired insulin signaling (Rehman et al., 2017). PKB is coordinate in the insulin cascade pathway, and its downregulation can cause IR and T2D. Stilbenes inhibit the overexpression of IL‐6 via downregulation of NF‐κB and activator protein‐1 (AP‐1) in many types of immune/inflammatory cells, fibroblasts, and endothelial cells (Yanxiang Li, Yang, He, et al., 2017). Moreover, IL‐6 facilitated the enhanced expression of JAK2/STAT3 is also down‐regulated by resveratrol.

### 4.5 Innate and adaptive immunity

Cytokine producing CD4+ and CD8+ T cells are differentiated, activated, and proliferated by anti‐CD3/anti‐CD28 stimulation. Then, different subsets of both CD4+ and CD8+ T cells produce various cytokines, involved in the regulation of different patterns of immunity. Type 1 produces IL2, IFN‐g, and lymphotoxin and is involved in cell‐mediated inflammatory response, while type 2 produces IL4, IL5, IL10, and IL13 and produces an antibody‐mediated immune response. T cells produce a significant amount of type 1 and 2 when stimulated via CD3 and CD28. In vitro resveratrol treatment has biphasic action on anti‐CD3/anti‐CD28. This inhibits the proliferation of CD4+ and CD8+ T cells and its subsequent cytokine release at higher concentrations of 10µg/ml (Lai et al., 2016).

### 4.6 Fatty acids homeostasis

Fatty acid dysregulated homeostasis is a significant predictor of obesity, IR, and T2D. Saturated fatty acids are crucial, and they accumulate and release several adipokines and chemokines which induce low grade, chronic, and systematic inflammation and hinder the action of insulin action (Rodriguez et al., 2018). Saturated fatty acids can also bind to TLR2 and TLR4 which turns on κB and JNK pathways. Resveratrol has a crucial role in the upregulation of pAMPK which further modulates the expression of AMPK and Sirt1. AMPK has an important role in the homeostasis of cellular energy. Sirt1 induces lipolysis and subsequently causes fat loss. Sirt1 also upregulates peroxisome proliferator‐activated receptor‐gamma coactivator 1 alpha (PGC1α), which is comprehensively described as the master regulator of free fatty acid oxidation and gluconeogenesis(Ran et al., 2017). An important structural membrane scaffolding protein called Caveolin‐1 has a prime role in cellular cholesterol homeostasis and fat transportation through autophagy‐mediated mechanisms. Caveolin‐1 upregulation has been noted in resveratrol treated cells (Chen et al., 2015).

### 4.7 Control of endoplasmic reticulum stress

Endoplasmic reticulum stress (ERS) is an essential element in the progression of IR and T2D. ERS induces inflammation and dysfunction in adipose tissue and the production of adipokines. In islets cells, ESR enhances β cell apoptosis consequently declining secretion of insulin. Similarly, in hepatocytes, it impairs gluconeogenesis and mediates IR (Villalobos‐Labra et al., 2019). Activating transcription factor 4 (ATF‐4) and Tribbles Pseudokinase 3 (TRIB3) are highly linked to ERS induced IR (Ko et al., 2013; J. Yang et al., 2016). A study directed by Zhao et al. (2019) indicated that hepatic expression of TRIB3 and AFT‐4 reduced in vitro when treated with resveratrol 20 μM. Expression of ATF‐6 enhanced which has been linked to improved insulin sensitivity via lowering ERS incidence. Other factors involved in the pathophysiology of ER stress are protein kinase‐like ER kinase (PERK) and eukaryotic initiation factor 2 alpha (eIF2 alpha). Activation of both these factors is strongly linked to the onset of ER stress. Results of many in vitro and in vivo researches have pointed that resveratrol has a strong impact on the downregulation of PERK and thus preventing the ER stress and consequently IR (Yuan et al., 2018; Zhao et al., 2019).

### 4.8 Regulation of mitochondrial dysfunction

During the excess supply of fatty acids and glucose, there is an increase in mitochondrial activity. Transmission of electrons during ETC produces superoxides which cause oxidative stress and potential downregulation of Nrf2 (Montgomery & Turner, 2015). In mitochondrial dysfunction, there is an overall decrease in substrate oxidation, mitochondrial content, and lower mitochondrial oxidative protein expression in response to which there is an increase in DAG, TAG, ceramides, and glucose level which are linked to IR. DAG translocates to the cell membrane through protein kinase C and hinders the action of insulin receptors. Ceramide causes inhibition of Akt (Rebollo‐Hernanz et al., 2019). The defensive role of resveratrol against mitochondrial dysfunctioning is through the upregulation of heme‐oxygenase‐1 (HO‐1). It causes downregulation of the p38 MAPK pathway which acts by modulating NFκB. NFκB is responsible for TNF‐α and IL‐1β release which causes inflammation. Resveratrol 100 μM for 1 hr counters the production of ROS/RNS via HO‐1. HO‐1 causes upregulation of catalase (CAT), superoxide dismutase (SOD), and glutathione peroxidase (GPX) (Bellaver et al., 2016). Alternatively, resveratrol controls mitochondrial biogenesis is through SIRT1 activation which promotes the deacetylation of peroxisome proliferator activator gamma coactivator 1 alpha (PGC‐1α), which is referred as a master regulator of mitochondrial biogenesis (Ungvari et al., 2011).

## 5 FUTURE DIRECTIONS

IR is increasing rapidly worldwide and the leading cause of T2DM. Biguanides and thiazolidinediones are currently being used as drugs against IR with potential side effects. So, it is important to find alternate pharmaceuticals with more effective results and less toxicity issues. Stilbenes are found commonly in dietary sources and have a prominent role against IR as discussed previously in detail. Stilbenes have a potential anti‐IR effect via various cellular and subcellular mechanisms including GLUT4 upregulation, insulin receptors sensitization, and lowering inflammation. This improves the action of insulin and alleviating T2DM. This makes stilbenes a favorable candidate as good replacement therapy for IR treatment in future. However, data from human studies are lacking and thus clinical trials should be highly encouraged in this regard.

## 6 CONCLUSION

IR is a multifactorial disorder characterized by hyperinsulinemia and hyperglycemia leading to T2D, and its cases are rising rapidly worldwide due to poor lifestyle practices. Phytochemicals like stilbenes are promising options to ameliorate this disparity while avoiding the side effects of pharmaceuticals. Stilbenes are a group of natural polyphenols possessing many health benefits and have shown prominent results to overcome IR in many animals model studies and cell line studies. Stilbenes act as an insulin‐sensitizing agent and prevent β‐cell loss. It also selectively activates the IRS1/PI3‐ k/Akt signaling pathway thus improving insulin signaling cascade and inhibiting IR. However, further studies are needed to elucidate their in‐depth mechanisms and to adjust their dose‐dependent effects in humans based on personalized diet plans or nutraceutical responses. Alongside, nutrigenetic studies are also required to evaluate the personalized effects of stilbenes for appropriate use in humans.
